# The Need for Specialized Pediatric Cardiac Critical Care Training Program in Limited Resource Settings

**DOI:** 10.3389/fped.2018.00059

**Published:** 2018-03-14

**Authors:** Indah K. Murni, Ndidiamaka L. Musa

**Affiliations:** ^1^Department of Pediatrics, Dr Sardjito Hospital, Universitas Gadjah Mada, Yogyakarta, Indonesia; ^2^Division of Pediatric Critical Care Medicine, Department of Pediatrics, University of Washington, Seattle Children’s Hospital, Seattle, WA, United States

**Keywords:** cardiac critical care, cardiac intensive care, training program, pediatrics, limited resources settings, low- and middle-income countries

## Abstract

Congenital heart disease (CHD) is one of the major global health problems with the highest birth prevalence in low- and middle-income countries. In these populous countries, basic health services for the children with CHD, including surgery, are lacking. Even though surgery is performed, outcome after cardiac surgery is influenced by the quality of the postoperative management with a reported high morbidity and mortality. Henceforth, there is an urgent need for comprehensive interventions to provide high quality cardiac intensive care programs to improve the quality of pediatric cardiac surgery services in order to address high morbidity and mortality after cardiac surgery. The development and training of the health workers in the field of pediatric cardiac intensive care program is required. It is imperative to conduct this training prior to actual implementation of the program in limited resources settings.

## Introduction

Congenital heart disease (CHD) is the most common cause of major congenital anomalies representing a major global health problem. The number of children with CHD is increasing worldwide. The increase might be caused by changes in diagnostic approaches and screening methods rather than representative of a true increase. The highest birth prevalence of CHD was reported from Asia. Although, this may be skewed by data from high-income countries in the region exclusively reporting the highest CHD prevalence, while no data were available from low-income countries in the region (1).

Approximately 1/100 babies worldwide are born with CHD, of which one fourth are critical CHD (1). Those with hemodynamically significant CHD would die in the first year of life without cardiac surgery. In populous low- and middle-income countries, basic health services for children with CHD, including surgery, are lacking. In addition, even when surgery is performed, outcome after surgery is influenced by the quality of the postoperative management of the patients. Mortality and morbidity after cardiac surgery have been widely used as measured indicators to evaluate the quality of pediatric cardiac surgical care (2). Mortality after surgery significantly differs depending on the complexity, severity of CHD and existing comorbidities (such as malnutrition and chronic infections). Previous studies in low- and middle-income countries found that mortality among children after cardiac surgery were deemed to be high: Indonesia (12.8%), Iran (12.4%) (3), Guatemala (10.7%) (4), India (7.9%) (5), and China (5.5%) (6). In contrast, in high-income countries, mortality after cardiac surgery in children has been reported to be less than 5% (7). In low- and middle-income countries, the outcomes of the pediatric cardiac surgery were impacted significantly by a number of factors that include limitations of human resources, inadequate facilities for cardiac surgery, and postoperative care management. While a lot has been done to establish pediatric cardiac surgical programs in low- and middle-income countries, there is now recognition that team-based training is essential for improved outcomes (8). This chapter focuses on addressing pediatric cardiac intensive care training in low- and middle-income countries.

## Pediatric Cardiac Critical Care (PCCC) in Low- and Middle-Income Countries

Factors related to morbidity and mortality after cardiac surgery and cardiac intensive care include high proportions of late presenters, major comorbidities, and severe complications of CHD. Accessibility to echocardiograms has highlighted the global inequalities of access to care. In high-income countries prenatal diagnosis has led to early detection of CHD before birth resulting in early diagnosis of lesions that could result in early referral and treatment long before the onset of irreversible sequel. This is not the case in low- and middle-income countries, where only a few children with CHD are even detected after birth. The proportions of children with missed diagnoses differ among centers, are considered to be much higher in low- and middle-income countries compared to high-income countries. A general lack of awareness and recognition among health care workers, lack of resources, and low numbers of properly trained healthcare workers could be considered as the underlying reasons for the delays. Majority of pediatricians receive little or no training in pediatric cardiology because there are very few pediatric cardiac programs in low- and middle-income countries (9). Furthermore, lack of pediatric cardiologists, skilled personnel and limitations in the existing infrastructure for cardiac surgery, and post-cardiac surgical care contribute significantly to the delayed diagnosis of CHD (7).

Cardiac critical care issues may affect a low resource setting in many ways. The use of cardiopulmonary bypass in children undergoing cardiac surgery may impact organ systems including altered vasomotor tone, fluid shifts, reduced pulmonary function, acute kidney injury, gastrointestinal, neurologic, hematologic endocrine dysfunction, and infection (10). Children admitted to intensive care unit (ICU) may be vulnerable to hospital-acquired infections. Patients who develop nosocomial bloodstream infections have significantly higher risk of mortality than those who do not (11, 12).

Among those who present with the late diagnosis of CHD, fewer options of beneficial surgical procedures are available due to complications at the time of diagnosis. Some of the most common complications among those who present with delayed diagnosis of CHD include congestive heart failure, severe pulmonary hypertension, severe malnutrition, chronic infection, severe polycythemia, chronic hypoxia, and stroke (13, 14).

For many children correctable surgeries are not performed in low-income countries because of the absence of trained congenital heart surgeons, limitations of human resources, inadequate facilities for cardiac surgery, and severe condition at the onset of diagnosis. On the other hand, if correctable surgery is performed the perioperative period is more hazardous because of complications related to delayed surgery and postoperative care. Management of these sequelae due to the late presentation are challenging and requires a comprehensive and multidisciplinary approach focusing on the complexity of CHD, therapeutic options, surgical complexity and postoperative management. Most patients require nutritional rehabilitation, control of heart failure management of pulmonary hypertension, prevention of hypoxic spell, treatment of infective endocarditis, and correction of anemia (13, 14). There is a need for multidisciplinary approach to the patient, which requires significant training of the individual members of the team.

## The Need for PCCC in Low- and Middle-Income Countries

The first step is recognition of some of the contributing factors as outlined above. There is a dire need for comprehensive interventions to provide high quality cardiac intensive care programs and to improve the quality of pediatric cardiac surgery services in order to address morbidity and mortality after cardiac surgery. Problems related to pediatric cardiac surgery and cardiac intensive care program in low- and middle-income countries are multifactorial and include competing priorities, poor structural organizations, lack of financial resources, lack of trained human resources, insufficient training, and educational infrastructure (7). In addition, there is increasing realization that the lack of facilities for sustainable pediatric cardiac services in low- and middle-income countries results in a massive number of preventable deaths and ongoing suffering (15). Henceforth, there are important considerations in addressing this burden, which include the prioritization of training a multidisciplinary team specializing in the care of the pediatric cardiac patient. This includes pediatric cardiac focused nurses, pediatric cardiologist, pediatric cardiac intensive care specialist or pediatric intensivists, anesthesiologists, and cardiothoracic surgeons. A team-oriented focus allows all the members to have intellectual ownership of the heart program (16).

However, to achieve these ideals in the setting of limited resources is challenging but is not impossible. These require an effective policy response, adequate financial investment to increase the numbers of cardiac intensive care programs and thereby improve the quality of pediatric cardiac surgery in low-and middle-income countries.

## Impact of PCCC Training Program in Low- and Middle-Income Countries

Pediatric cardiac critical care (PCCC) training programs are very few in low- and middle-income countries (17). Henceforth, the impact of PCCC training program is difficult to assess. There are few published studies evaluating the impact of PCCC and a few studies were found evaluating the impact of the presence a dedicated pediatric cardiac intensive care (16, 18). However, there were no randomized controlled trials with opportunity of providing robust evidence. Since it is very challenging to do a randomized controlled trial in patients with CHD, a methodological sound quasi-experimental study to determine the effectiveness of pediatric cardiac intensive care training program would be feasible and practical in clinical settings.

In high-income countries, several published studies evaluating the impact of a dedicated pediatric cardiac intensive care unit on the outcome among children undergoing cardiac surgery for CHD has demonstrated better outcomes in lowering bloodstream infections, preventing prolonged use of mechanical ventilation, and reducing length of ICU stay (19–21). A systematic review in adults showed that the greater use of intensivist in adult ICUs led to a significant reduction in ICU and hospital mortality towards length of stay (22).

Henceforth, the development and training of the health workers in the field of pediatric cardiac intensive care is required and if possible should be put in place prior to actual implementation of a program in limited resources settings.

## Challenges and Possible Solutions of PCCC Training Program in Low- and Middle-Income Countries

Challenges occur during the development, implementation, and maintenance phases of a PCCC training program. The challenges consist of the availability and maintenance of equipment in pediatric cardiac intensive care unit, including invasive monitoring, life saving intervention, renal replacement therapy, and hemodynamic monitoring. The possible solutions that are relevant to limited resource settings include reducing costs; in addition availability of medical equipment should be appropriate to available resources and needs (23).

Due to limited infrastructure, funding, and human resources in low- and middle-income countries, a dedicated pediatric cardiac intensive care is not widely available as compared to high-income countries. The cardiac services are provided by a multidisciplinary team including pediatric cardiologists, pediatric cardiac surgeons, intensivists, anesthesiologists, critical care nurses, respiratory therapists, and other allied health workers who are available to care for children after cardiac surgery (21, 24, 25).

The team leader of the pediatric cardiac intensive care unit could be a pediatric cardiologist who has an experience or training in intensive care, a pediatric intensivist who has training or knowledge on hemodynamics of CHD and management of children after cardiac surgery, anesthesiologist, or adult intensivist with adequate knowledge in caring for neonates, infants, or children who have frequently been involved in pediatric cardiac surgery (4, 18). Pediatric cardiac intensive care in low- and middle-income countries are few and far between. Various models exist either through a specialized pediatric cardiac intensive care facility in a tertiary hospital, or as a part of a general pediatric or even adult ICU.

There are considerable gaps in providing an ideal environment to care for children after cardiac surgery in low- and middle-income countries. Henceforth, emphasis should be placed on the need for a comprehensive and multidisciplinary approach to the management of pediatric cardiac patients regardless of their location. The cost of providing care in a dedicated pediatric cardiac intensive care may be prohibitive when resources are limited. However, collaborations with well-established programs in high resource settings has led to an evolution in developing pediatric cardiac intensive care units in low- and middle-income countries (16). The partnership with non-governmental organizations has led the way in demonstrating that these barriers in training and organization can be prevailed.

There are challenges to training and building a sustainable PCCC program in resource limited settings. Education and training are very important to create physician and nursing competency in the care of children with critical cardiac conditions. There are wide ranges of training experience on postoperative patients that are provided in high-income countries. Sub-specialization from high-income countries has evolved over time. In recent years majority who practiced this specialty have additional and formal training (26, 27). This is challenging for those in limited resource settings where there is a shortage of healthcare personnel working in PCCC setting.

Training in PCCC in low- and middle-income countries has evolved over the time which is variable depending on the country and the visiting teams. Teams from well-established PCCC program in high-income countries have been important drivers in teaching and building the host teams confidence and experience (28). Cardiac surgical training programs have used a variety of methods that we could learn from such as short-term observerships which are not adequate for skill acquisition and maintenance, fellowship training sometimes non-accredited have been the most productive in the past. Recently, imbedding projects where experienced cardiologists or surgeons spend 1–12 months in the host programs has proven to be medically and financially valuable (28). Telemedicine is advancing and can be a useful tool for consultation but not for training. Furthermore, a model of mentoring and supporting physicians and other staff from well-established cardiac centers in high-income countries through partnerships with programs in low-middle income countries should be prioritized. The international quality initiative collaborative on CHD highlights the fact that through collaboration, training, nursing empowerment and benchmarking care can be improved even in low- and middle-income countries (29). These strategies are important to help substantially, when a new program is being established with limited resource settings.

Retention of the trained staff working in limited resource settings is a key to the sustainability of a developing program. Factors that have led to the migration of staff can be prevailed. This might be prevailed by hiring the right candidates at the beginning or providing a decent salary. Creating or sustaining a structured program with committed personnel to surround new personnel might help to ensure an ongoing staff development (26).

Indonesia just like other developing countries still struggles with high mortality rate, even with its many significant achievements on societal, political, and economic development. Referral in neonatal and pediatric emergencies is crucial due to the unpredictability of the complications and their potential to progress rapidly to become severe and life threatening. Deaths could be prevented if referral systems were in place and functional to allow them to reach appropriate health services to prevent complications. The healthcare system must be adequate to respond to referral patients in a timely manner and with quality care in order to save the lives of those referred.

These would be very challenging for Indonesia, a country with a population of over 250 million and has long distances between each other islands, only has six institutions with comprehensive facilities to take care of the children with CHD. The substantial improvement in the number of high quality comprehensive cardiac services is needed.

In order to maintain the sustainability of the program, a Steering Committee should be established prior to the commencement of the training program to seek their support and endorsement of the intervention. This includes all the stakeholders, hospital director, the head of departments, nursing, cardiac surgeon, pediatric cardiologist, intensivist, anesthesiologist, nutritionist, physiotherapist, pharmacist and other clinical, and allied health staff. The proposed program should be presented to seek their support and endorsement of the program. The Steering Committee should be asked to review and provide feedback about the available information on the development and maintenance phases that will be used during the training program. Further, all the healthcare personnel who are caring for patients after cardiac surgery should be asked to participate in a quality improvement initiative.

## Conclusion

Published studies on the pediatric cardiac intensive care program and its effectiveness in limited resource settings are very few. With increased demand of proper management for CHD in such settings, the need of PCCC training program is unavoidable and should be prioritized to improve outcome. In addition, impact of this training program should be evaluated to determine the performance and outcomes of children with heart disease in low- and middle-income countries.

## Author Contributions

IM and NM determined the review question. IM wrote the first drafts of the paper. IM and NM contributed to the reviewing and interpretation of results, writing of the paper, had input and approved the final version.

## Conflict of Interest Statement

The authors declare that the research was conducted in the absence of any commercial or financial relationships that could be construed as a potential conflict of interest.
